# Tolerance and Heavy Metal Accumulation Characteristics of *Sasa argenteostriata* (Regel) E.G. Camus under Zinc Single Stress and Combined Lead–Zinc Stress

**DOI:** 10.3390/toxics10080450

**Published:** 2022-08-04

**Authors:** Jiarong Liao, Ningfeng Li, Yixiong Yang, Jing Yang, Yuan Tian, Zhenghua Luo, Mingyan Jiang

**Affiliations:** College of Landscape Architecture, Sichuan Agricultural University, Chengdu 611130, China

**Keywords:** combined zinc–lead stress, *Sasa argenteostriata* (Regel) E.G. Camus, accumulation characteristics

## Abstract

*Sasa argenteostriata* (Regel) E.G. Camus is a gramineous plant with the potential for phytoremediation. In this study, we aimed to determine its tolerance to zinc stress and combined lead–zinc stress and the effect of zinc on its absorption and accumulation characteristics of lead. The results showed that *S. argenteostriata* had good tolerance to zinc stress, and *S. argenteostriata* was not significantly damaged when the zinc stress concentration was 600 mg/L. Under both zinc stress and combined lead–zinc stress, the root was the main organ that accumulated heavy metals in *S. argenteostriata*. The presence of zinc promoted the absorption of lead by the root of *S. argenteostriata*, and the lead content in the root under PZ1, PZ2, PZ3 and PZ4 treatments was 2.15, 4.31, 4.47 and 6.01 times that of PZ0 on the 20 days. In the combined lead–zinc stress treatments, the toxicity of heavy metals to *S. argenteostriata* was mainly caused by lead. Under high concentrations of combined lead–zinc stress (PZ4), the proportion of zinc in the leaf of *S. argenteostriata* on the 20 days increased, which was used as a tolerance strategy to alleviate the toxicity of lead.

## 1. Introduction

With the rapid development of the mining industry, many lead (Pb) and zinc (Zn) mining areas have been over-exploited, which has caused serious pollution to the surrounding soil and environment [1,2]. When pollution occurs, Pb and Zn are typically associated with one another, causing joint hazards such as increased potential bioavailability and the crop accumulation of Pb and Zn [3]. This causes serious dietary risks to residents, especially children, around the mining area [4,5].

Among the many restoration methods, phytoremediation has attracted increasing attention due to its sustainability, low cost and environmental friendliness [6,7]. Most plants with Pb–Zn remediation potential have been found by collecting and measuring the plant samples around Pb and Zn mining areas. Stefanowicz et al. (2016) showed that *Fragaria vesca*, *Potentilla arenaria*, *Plantago lanceolata* and *Scabiosa ochroleuca* have a relatively high plant/soil Pb–Zn content ratio and a relatively low shoot/root Pb–Zn content ratio [8], which can be useful in phytostabilization. Twenty-five plant species collected by Chaabani et al. (2017) were found to be tolerant to both Pb and Zn and are found as volunteers in contaminated soil [9]. However, the plants around the mining area in the current study generally have insufficient biomass and slow growth; therefore, they cannot be widely used in practice [10,11]. Thus, it is necessary to determine tolerant plants with high biomass and fast growth in non-mining areas.

Numerous studies have been conducted on the screening of plants in non-mining areas under the combined stress of Pb and cadmium (Cd) or Zn and Cd. Most studies have shown that the toxicity of combined Pb–Cd or Zn–Cd stress was stronger than that of single heavy metal stress [12,13,14,15,16,17,18]. When combined, the absorption and transport of various heavy metals by plants have no obvious regularity. Liu et al. (2008) investigated the effect of Cd single stress and combined Pb–Cd stress on three plant species, indicating that the interaction of heavy metals in plants is complex and related to many factors such as heavy metal species, heavy metal concentration and plant species [19]. Under combined Cd–Zn stress, root development and plant growth are restricted [16,17,18], which may be attributed to the increase in the total concentration of metals. However, as an essential element in plants and similar to Cd, Zn can reduce the toxicity of Cd to plants to a certain extent [20]. Currently, the mitigation of Zn to Cd toxicity is due to Cd dilution caused by Zn competition [21]. However, studies on combined Pb–Zn stress are still rare.

Bian et al. (2020) showed that bamboo is an untapped plant resource with the potential for heavy metal remediation [22]. *Phyllostachys pubescens*, *Pleioblastus fortunei*, *Indocalamus decorus* and *Sasa argenteostriata* (Regel) E.G. Camus have been reported to have a strong remediation effect on Pb, Zn, Cd and other heavy metals [23,24,25,26,27]. For *S. argenteostriata* under Pb stress, there is the detoxification strategy of the transformation of Pb to a low-toxicity/low-migration form [28], the tolerance strategy of the root absorbing a large amount of Pb and isolating it in vacuoles [29] and the effective resistance mechanisms of inducing the antioxidant enzyme system and ascorbic acid-glutathione cycle and promoting chelation [30]. However, the tolerance and accumulation characteristics of *S. argenteostriata* to Zn and Pb–Zn are still unclear. This study investigates the tolerance of *S. argenteostriata* to Zn stress and combined Pb–Zn stress. In addition, this study investigates the interaction of Pb and Zn in the absorption and transport process of *S. argenteostriata* to explore the potential application of *S. argenteostriata* in the phytoremediation of combined Pb–Zn stress.

## 2. Materials and Methods

### 2.1. Plant Materials and Culture

The materials used in the experiment were biennial breeding seedlings obtained from the Garden Ecological Experiment Base of Sichuan Agricultural University (30°42″N, 103°51″E and 535 m.a.s.l). *S. argenteostriata* individuals with the same growth status were selected, and the rhizosphere soil was removed by washing with water. Fifteen plants were planted in each opaque, 10 L hydroponic barrel containing a 5 L nutrient solution (2000 times dilution, pH 5.0–5.5). The composition of the nutrient mother solution and maintenance were previously described by Jiang et al. (2020) [28]. The nutrient solution was replaced every 4 days, and a stress test was carried out after 20 days of pre-culture.

### 2.2. Experimental Design

#### 2.2.1. Zn Single-Stress Treatment

Pre-experiments showed that, when the Zn concentration was 250, 500 and 1000 mg/L, the degree of toxicity was not obvious, obvious and very serious, respectively. Therefore, the medium and low-stress concentrations (0, 75, 150, 300 and 600 mg/L) were increased, and the upper limit of stress concentration (1000 mg/L) was retained in the formal experiment. Briefly, six Zn stress treatments were designed. Zn concentrations were 0, 75, 150, 300, 600 and 1000 mg/L and are denoted as Z0, Z1, Z2, Z3, Z4 and Z5 throughout the text. Zn was calculated as pure Zn and was added as Zn(NO_3_)_2_. Except for the trace amount of Zn^2+^ in the nutrient solution (negligible), no additional Zn was added to the control (Z0). The first sampling time was when the plant showed slight growth inhibition (4–5 leaves of the plant withered and turned yellow under the highest stress concentration); the second sampling time was when plants showed strong growth inhibition (more than half of the plant leaves withered and turned yellow under the highest stress concentration).

#### 2.2.2. Combined Pb–Zn Stress Treatment

A previous study found that the Pb concentration of 300 mg/L was a moderate and fully tolerated concentration for *S. argenteostriata* [28]. In order to study the tolerance and accumulation characteristics of heavy metals of *S. argenteostriata* after superimposing Zn, this study adopted an experimental design with a fixed Pb concentration and an increasing Zn concentration. Referring to the Zn single-stress study, six combined stress levels were designed. The concentrations of Pb–Zn were 0–0, 300–0, 300–75, 300–150, 300–300 and 300–600 mg/L and are abbreviated as CK, PZ0, PZ1, PZ2, PZ3 and PZ4, respectively. Pb and Zn were calculated as pure Pb and pure Zn and were added as Pb(NO_3_)_2_ and Zn(NO_3_)_2_. The application of Zn and the collection of samples followed the methods previously described in 2.2.1. Samples in the combined Pb–Zn stress treatment were collected on the 10th and 20th days after the initiation of the stress treatment.

### 2.3. Determination of Plant Physiological Indexes

Fresh plant samples from both Zn single stress and combined Pb–Zn stress were collected twice, with the physiological indexes measured only once at the instance of first collection. The determination of physiological indexes reflected the damage of heavy metal stress to plants and the tolerance of plants to it. The roots were washed with 20 mM EDTA-Na_2_ solution and deionized water. The leaves were washed with deionized water, and the midrib was removed. They were cut into pieces, mixed, weighed at 0.2 g and then wrapped in tin foil. They were quickly frozen with liquid nitrogen and stored at −80 °C. The content of malondialdehyde (MDA) in roots and leaves was determined using the thiobarbituric acid method [31]. In order to better reflect the physiological tolerance of plants, the free proline (Pro) content and soluble sugar (SS) content of leaves were determined. The content of Pro in leaves was determined following the acid ninhydrin colorimetric method [32]. The content of SS in leaves was determined using the anthrone colorimetric method [33]. The above indexes were measured by a Multiskan GO full-wavelength reader (Model Thermo Scientific, Waltham, MA, USA).

### 2.4. Determination of Metal Content in Plant Tissues

The metal contents of plant tissues were measured twice in both the Zn single stress and combined Pb–Zn stress. The first determination reflected the accumulation characteristics of plants to heavy metal; the second determination of metal content in plant tissues reflected the maximum accumulation ability of plants to heavy metal. Bamboo seedlings were collected and divided into four organs: root, rhizome, stem and leaf. The plant samples were washed with deionized water after removing the ions that adhered to the surface of the root and rhizome with the 20 mM EDTA-Na_2_ solution. The samples were dried in an oven at 105 °C to a constant weight. After grinding by a stainless-steel grinder, the samples were passed through a 100-mesh nylon sieve for later use. After the 0.2 g dry sample was digested with HNO_3_-HClO_4_ (*v*/*v*, 5:1), the contents of Pb and Zn in the sample were determined by atomic absorption spectrometry (FAAS-M6, Thermo Fisher Scientific, Waltham, MA, USA). The bioaccumulation factors (BCFs) of heavy metals are defined as the proportional constants between the heavy metal concentrations in plant tissues and that in the environment [34]. The formula used to calculate BCFs is as follows, where C represents the concentration of heavy metals (mg/kg):

Bioaccumulation factor (BCF) = C_(plant tissue, mg/kg)/_C_(treatment media, mg/L)_

### 2.5. Statistical Analysis

All data were analyzed for a one-way ANOVA using SPSS (ver. 23: SPSS Inc., Chicago, IL, USA). Data from different times under the same treatment were compared using a Tukey test at *p* < 0.05 and *p* < 0.01. Excel 2010 (Microsoft Inc., Redmond, WA, USA) was used for graphing and data analysis.

## 3. Results and Analysis

### 3.1. Responses of Stress-Resistance Physiology and Heavy Metal Accumulation Characteristics of S. argenteostriata under Zn Stress

**Figure 1:**
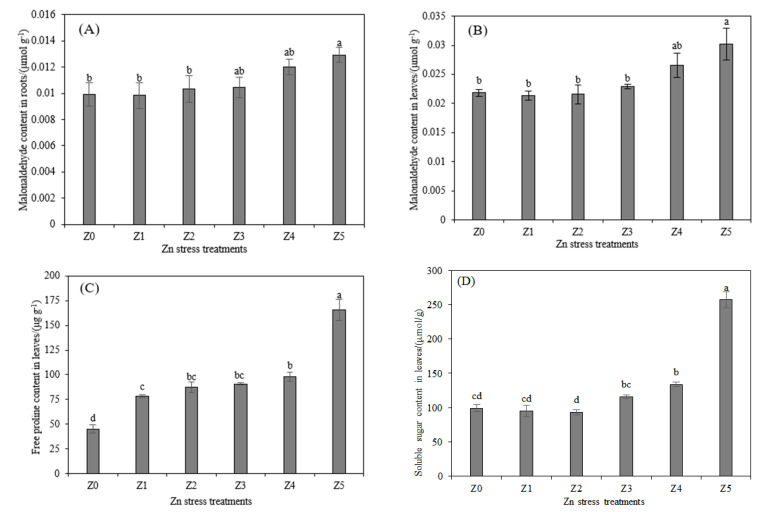
Impacts of different Zn treatments on malonaldehyde content in roots (**A**), malonaldehyde content in leaves (**B**), free proline content in leaves (**C**) and soluble sugar content in leaves (**D**). Along the *x*-axis of each graph, Z0, Z1, Z2, Z3, Z4 and Z5 indicate that the Zn concentrations are 0 (or control), 75, 150, 300, 600 and 1000 mg/L, respectively. Vertical bars represent the standard errors of the mean (*n* = 3). Different letters indicate significant differences among treatments with different Zn concentrations (*p* < 0.05).

The data presented in Figure 1A,B indicate that, only in the Z5 treatment, the MDA content in the roots and leaves of *S. argenteostriata* was significantly different from that of the Z0 (*p* < 0.05), indicating that 600 mg/L is an acceptable concentration of Zn stress for *S. argenteostriata*, whereas *S. argenteostriata* was negatively impacted when the concentration reached 1000 mg/L. The content of Pro and SS also followed a similar trend. Compared with Z4, the content of Pro and SS under Z5 showed the greatest increase (68.7% and 92.1%). In addition, the Pro content of *S. argenteostriata* under Z1 was significantly increased relative to Z0 (*p* < 0.05). The SS content of *S. argenteostriata* was not significantly different from Z0 in the Z1, Z2 and Z3 treatments (*p* > 0.05), but the SS content of *S. argenteostriata* in the Z4 treatment was significantly greater than that in Z0 (*p* < 0.05).

**Figure 2:**
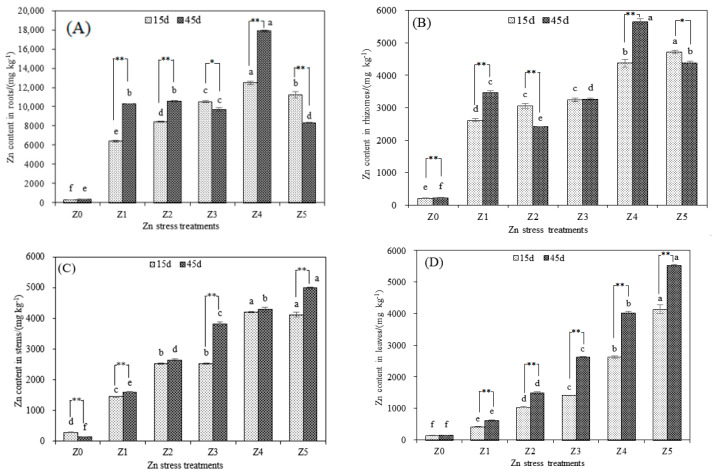
Impacts of different treatment times and Zn treatments on Zn content in roots (**A**), Zn content in rhizomes (**B**), Zn content in stems (**C**) and Zn content in leaves (**D**). Along the *x*-axis of each graph, Z0, Z1, Z2, Z3, Z4 and Z5 indicate that the Zn concentrations are 0 (or control), 75, 150, 300, 600 and 1000 mg/L, respectively. Vertical bars represent the standard errors of the mean (*n* = 3). Different letters indicate significant differences among treatments with different Zn concentrations under the same treatment time (*p* < 0.05). * indicates a significant difference at 0.05 under the same treatment and different time (*p* < 0.05). ** indicates an extremely significant difference at 0.01 under the same treatment and different time (*p* < 0.01).

The root is the primary location of the accumulation of Zn in *S. argenteostriata* under Zn stress conditions (Figure 2). On the 15th day, the Zn content in roots of *S. argenteostriata* in the Z5 treatment decreased by 9.86% compared to the Z4 treatment, and it decreased by 53.59% on the 45th day. The maximum Zn content in roots and rhizomes of *S. argenteostriata* was observed in the Z4 treatment 45 days after treatment initiation. However, at both 15 and 45 days, the Zn content in leaves increased significantly with the increase in Zn stress concentration (*p* < 0.05). In addition, the Zn content in stems and leaves increased with the increased treatment duration in treatments Z1, Z2, Z3, Z4 and Z5, especially in leaves.

At the same treatment time, with the increase in the Zn stress concentration, the Zn BCF of each organ of *S. argenteostriata* showed an overall decreasing trend (Table 1). In the Z5 treatment, with the increased duration of the treatment, the BCF of the root and rhizome of *S. argenteostriata* decreased, whereas the BCF of the stem and leaf increased. The BCF of the leaf was higher than that of the rhizome and stem at 45 d in the Z5 treatment.

### 3.2. Responses of Stress-Resistance Physiology and Heavy Metal Accumulation Characteristics of S. argenteostriata under Combined Pb–Zn Stress

**Figure 3:**
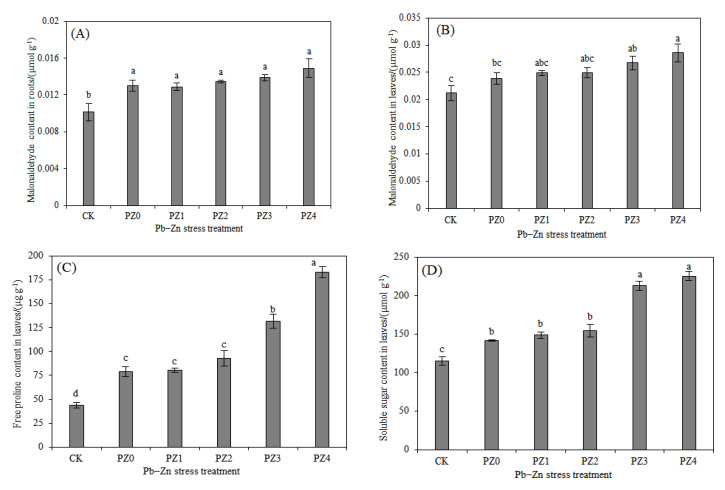
Impacts of different Pb–Zn treatments on malonaldehyde content in roots (**A**), malonaldehyde content in leaves (**B**), free proline content in leaves (**C**) and soluble sugar content in leaves (**D**). Along the *x*-axis of each graph, CK indicates that the Pb and Zn concentration is 0 (or control), and PZ0, PZ1, PZ2, PZ3 and PZ4 indicate that the Pb concentration are 300 mg/L and that the Zn concentrations are 0, 75, 150, 300 and 600 mg/L, respectively. Vertical bars represent the standard errors of the mean (*n* = 3). Different letters indicate significant differences among treatments with different Zn concentrations (*p* < 0.05).

There was no significant difference in MDA content in the roots in the PZ0, PZ1, PZ2, PZ3 and PZ4 treatments (*p* > 0.05), and only the MDA content in the leaves in the PZ4 treatment was significantly different from the PZ0 treatment (*p* < 0.05; Figure 3A,B). The results indicate that the increase in Zn stress concentration did not significantly increase the lipid peroxidation of the cell membrane. The two osmotic adjustment substances (Pro and SS) followed a similar trend. The Pro and SS contents in the PZ3 and PZ4 treatments were significantly higher than those in the PZ0, PZ1 and PZ2 treatments (*p* < 0.05), and there was no significant difference among the PZ0, PZ1 and PZ2 treatments (*p* > 0.05). However, when the Zn concentration was increased to 600 mg/L (PZ4), the SS content was no longer significantly increased relative to the PZ3 treatment (*p* > 0.05), but the Pro content was significantly increased by 39.26% (*p* < 0.05).

**Figure 4:**
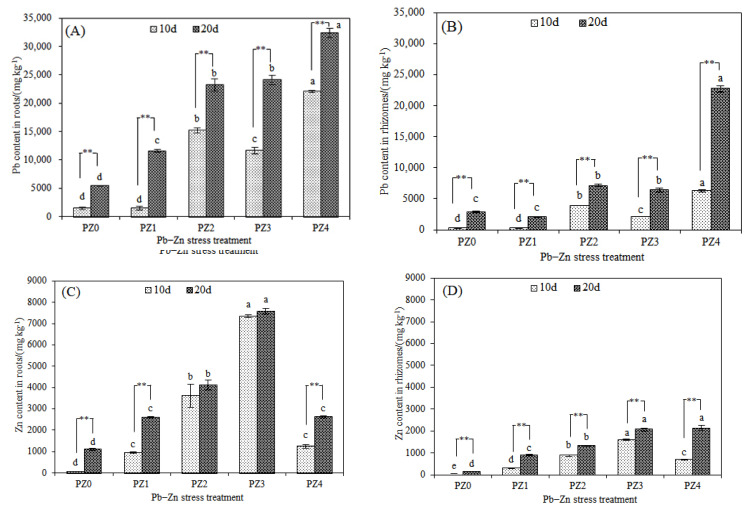

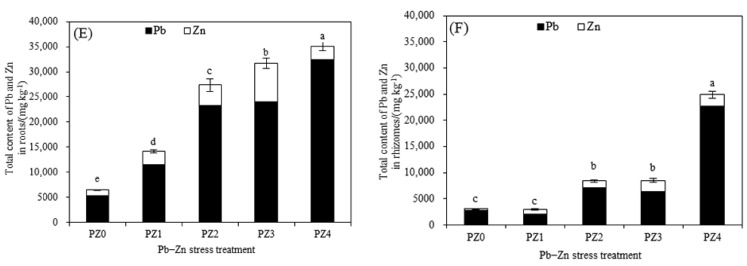
Impacts of different treatment durations and Pb–Zn treatments on Pb content in roots (**A**), Pb content in rhizomes (**B**), Zn content in roots (**C**), Zn content in rhizomes (**D**), the total content of Pb and Zn in roots after 20 days of treatment (**E**) and total content of Pb and Zn in rhizomes after 20 days of treatment (**F**). Along the *x*-axis of each graph, PZ0, PZ1, PZ2, PZ3 and PZ4 indicate that the Pb concentration is 300 mg/L and that the Zn concentrations are 0, 75, 150, 300, and 600 mg/L, respectively. Vertical bars represent the standard errors of the mean (*n *= 3). Different letters indicate significant differences among treatments with different Zn concentrations under the same treatment time (*p* < 0.05). ** indicates an extremely significant difference at 0.01 under the same treatment and different time (*p* < 0.01).

With the increased duration of stress treatment, both the Pb and Zn contents in the roots and rhizomes of *S. argenteostriata* increased (Figure 4A–D). On the 20th day of treatment, the Pb content in the roots in the PZ1, PZ2, PZ3 and PZ4 treatment was significantly higher than that in the PZ0 treatment (*p* < 0.05), and with the increase in Zn stress concentration, the Pb content in roots under all treatments also gradually increased (Figure 4A). In addition, on the 20th day, the Pb content in the rhizomes of *S. argenteostriata* in the PZ4 treatments was 3.63 times that on the 10th day, which was as high as 22,755.52 mg/kg (Figure 4B). The Zn content in the roots of *S. argenteostriata* showed a trend of first increasing and then decreasing with the increase in Zn stress concentration. Compared with PZ3, the Zn content in the roots of *S. argenteostriata* in the PZ4 treatment decreased by 83.19% and 65.30% at 10d and 20d, respectively (Figure 4C). The Zn content in the rhizomes also followed a similar decreasing trend (Figure 4D). Compared with the PZ3 treatment, the Zn content in the rhizomes in the PZ4 treatment was significantly decreased by 56.52% on the 10th day of treatment (*p* < 0.05), and there was no significant difference on the 20th day of treatment (*p* > 0.05). When treated for 20 days, the total contents of Pb and Zn in the roots and rhizomes of *S. argenteostriata* were mainly determined by the Pb content because the Zn content was relatively low (Figure 4E,F). The total content of Pb and Zn in the roots of *S. argenteostriata* increased significantly with the increase in Zn stress concentration (*p* < 0.05). In the PZ3 treatment, the Zn content in the roots accounted for the largest proportion of Zn, which was 23.93% of the total content (Figure 4E). In the PZ1 treatment, the Zn content in the rhizome accounted for the largest proportion of Zn, which was 30.13% of the total content (Figure 4F).

**Figure 5:**
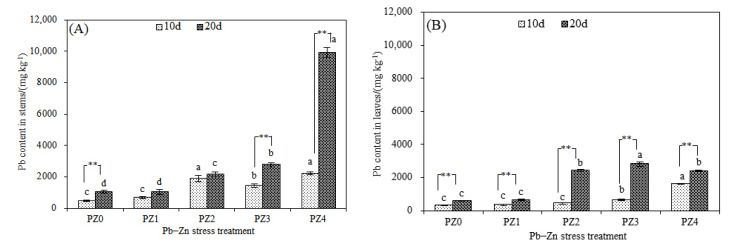

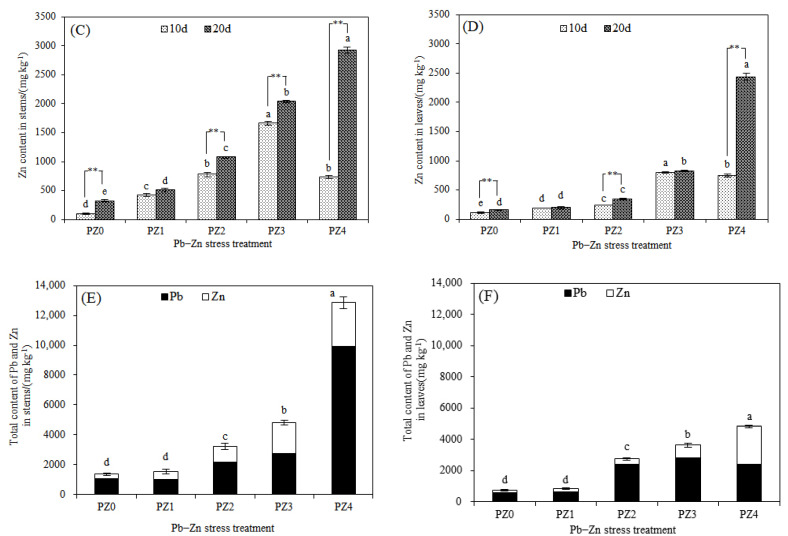
Impacts of different treatment times and Pb–Zn treatments on Pb content in stems (**A**), Pb content in leaves (**B**), Zn content in stems (**C**), Zn content in leaves (**D**), the total content of Pb and Zn in stems after 20 days of treatment (**E**) and total content of Pb and Zn in leaves after 20 days of treatment (**F**). Along the *x*-axis of each graph, PZ0, PZ1, PZ2, PZ3 and PZ4 indicate that the Pb concentration is 300 mg/L and that the Zn concentrations are 0, 75, 150, 300, and 600 mg/L, respectively. Vertical bars represent the standard errors of the mean (*n* = 3). Different letters indicate significant differences among treatments with different Zn concentrations under the same treatment time (*p* < 0.05). ** indicates an extremely significant difference at 0.01 under the same treatment and different time (*p* < 0.01).

Consistent with roots and rhizomes, the content of Pb and Zn in stems and leaves increased with the increased treatment time (Figure 5A–D). In the PZ4 treatment, the Pb content in the stems of *S. argenteostriata* on the 20th day increased greatly, which was 3.58 times that of PZ3 (Figure 5A). In addition, the Zn content in stems and leaves in the PZ4 treatment was significantly lower than that in the PZ3 treatment on the 10th day (*p* < 0.05) and significantly higher than that in the PZ3 treatment on the 20th day (*p* <0.05), with an increase of 42.94% and 195.22%, respectively (Figure 5C,D). When the treatment duration lasted for 20 days, the total content of Pb and Zn in stems of *S. argenteostriata* increased significantly with the increase in Zn stress concentration (*p* < 0.05). The proportion of Zn in stems in the PZ4 treatment decreased relative to that in the PZ3 treatment (Figure 5E; PZ3: 42.43%; PZ4: 22.74%). Unlike in the stems, the proportion of Zn in leaves increased with the increase in the Zn stress concentration, reaching 50.4% in the PZ4 treatment (Figure 5F).

According to the BCF of the total heavy metals, the heavy metal accumulation capacity of each organ of *S. argenteostriata* was roots > rhizomes > stems > leaves (Table 2). The Pb BCF of the roots and rhizomes was higher than that of the stems and leaves under the combined Pb–Zn stress. With the increase in Zn concentration, the Pb BCF of the roots gradually increased. However, the Zn BCF of roots and rhizomes decreased with the increase in Zn stress concentration. In the PZ4 treatment, the Zn accumulation capacity of all organs was stems > roots > leaves > rhizomes.

## 4. Discussion

### 4.1. Tolerance and Heavy Metal Accumulation Characteristics of S. argenteostriata under Zn Stress

When plants are stressed by heavy metals, Pro and SS act as osmotic-regulating agents to maintain the water balance of cells and tissues and to protect the structural integrity of cell membranes [35,36]. In addition to being a general marker of osmotic stress, Pro can also purify hydroxyl radicals and singlet oxygen to reduce cell damage [37,38]. For *S. argenteostriata*, when the Zn stress concentration reached 1000 mg/L, its roots and leaves were obviously damaged. *S. argenteostriata* synthesized a large amount of Pro and SS to maintain the stability of cell osmotic potential and reduced membrane lipid peroxidation. In our study, with the increase in Zn stress concentration, Pro showed a significant difference before a change in SS was observed, compared with Z0 (Figure 1; *p* < 0.05). These findings indicate that *S. argenteostriata* can effectively respond to Zn stress by synthesizing Pro.

Heavy metals are accumulated in different organs depending on the plant species. For example, the main organ of Zn accumulation in *Ricinus communis* was the root [39], and the main organ of Zn accumulation in *Origanum vulgare* was the leaf [37]. In our study, the roots and rhizomes were the main organs of *S. argenteostriata* that accumulated Zn. Under high concentrations of Zn stress (Z5), the absorption of Zn by roots of *S. argenteostriata* was inhibited, and the inhibition effect was strengthened with the increased study duration. The difference was that, under high concentrations of Zn stress (Z5), the uptake of zinc by the above-ground parts (stems and leaves) of *S. argenteostriata* was not significantly inhibited, and the zinc content in the stems and leaves increased with the increased study duration. This may be the self-protection mechanism of the roots, thus alleviating the toxicity of Zn by reducing the absorption of Zn [40] and upward transport of Zn. The specific mechanism needs to be further studied. It was worth noting that, as a unique organ of bamboo, the rhizomes of *S. argenteostriata* accumulated a large amount of Zn under Zn single stress. Bian et al. (2017) found that, in a natural polluted environment, the rhizomes of moso bamboo had a higher copper content (only second to the roots) and a lower Zn content [41]. Although the accumulation capacity of rhizomes was not as good as that of the roots, it had a large biomass [42]. The function of rhizomes should not be ignored when studying the remediation effect of bamboo to heavy metal.

### 4.2. Tolerance and Heavy Metal Accumulation Characteristics of S. argenteostriata under Combined Pb–Zn Stress

Zn is an essential trace element in plants, but excess affects the growth of the plant, physiology, etc. [43]. In our study, the increase in Zn concentration (constant Pb concentration) did not evidently enhance the membrane lipid peroxidation of *S. argenteostriata*, indicating that the toxicity of combined Pb–Zn stress to *S. argenteostriata* was mainly caused by Pb. With the increase in the Zn concentration, the Zn content in the leaves of *S. argenteostriata* increased gradually. In the PZ4 treatment for 20 days, the proportion of Zn content in the leaves of *S. argenteostriata* was greater than that of Pb. Therefore, we speculate that the leaves of *S. argenteostriata* preferentially absorb Zn as a tolerance strategy to alleviate the toxicity of Pb [21,44]. In addition, *S. argenteostriata* has been reported to be able to resist single Pb stress by greatly increasing Pro [29,30], also under combined Pb–Zn stress. Thus, we can know that the increase in Pro content is an effective way for *S. argenteostriata* to resist heavy metal toxicity.

Most studies have found that the roots are the main organs of heavy metal accumulation in plants [45,46,47]. In addition, previous studies have shown that the roots and rhizomes were the main organs for heavy metal accumulation in *S. argenteostriata* under Pb stress [23,28]. In our study, under the combined Pb–Zn stress, the underground organs (roots and rhizomes) of *S. argenteostriata* primarily accumulated Pb. The enrichment and migration of various metals in plants are not only affected by the properties and concentrations of a specific single element, but also by coexisting elements and their interactions [48]. Santos et al. (2020) found that Zn inhibits the absorption of Cd by the roots of juvenile *Theobroma cacao* [44]. Sbartai et al. (2012) found that, when the Zn stress concentration is higher than Cd, the absorption of Cd by the roots of *Lycopersicon esculentum* is inhibited [47]. Adamczyk-Szabela et al. (2020) found that the addition of Zn reduces manganese (Mn) content in the roots of *Melissa officinalis* [49]. It should be noted that most of the discharged Pb falls on soil, water and organisms, especially plants that grow along roads [50]. Pb cannot be easily absorbed by plants and have difficulty transferring it to the aboveground part of plants, and it is a very contaminated metal for vegetation [51,52,53,54]. Under high concentrations (PZ4) and long-term stress (20d), Zn accumulation in the aboveground parts (stems and leaves) of *S. argenteostriata* exceeded that in the underground parts (roots and rhizomes). For *S. argenteostriata*, the presence of Zn significantly promoted the absorption and accumulation of Pb by the roots, and the effect of the promotion increased with the increase in the Zn concentration.

## 5. Conclusions

Previous studies have found that *S. argenteostriata* has strong tolerance and accumulation to Pb single stress. However, the present pollution was mostly combined pollution, and Pb and Zn were typically associated with one another, causing joint hazards. In this study, a Zn single-stress test was conducted to determine whether *S. argenteostriata* had a good tolerance to Zn stress. The roots of *S. argenteostriata* were the primary organs accumulating Zn, and the absorption of Zn followed a trend of, when the concentration of Zn stress was low, the absorption of Zn by the roots increased with the increase in the concentration of Zn stress. When the Zn stress concentration was 600 mg/L, the Zn content in the roots of *S. argenteostriata* was as high as 17,883.33 mg/kg on the 45th day. Under combined Pb–Zn stress, the toxicity of heavy metals to *S. argenteostriata* was mainly caused by Pb. The roots were the main organs of *S. argenteostriata* that accumulated Pb. The presence of Zn could promote the absorption of Pb by the roots of *S. argenteostriata*, and the promotion was enhanced with increases in the Zn stress concentration. When the Pb–Zn stress concentration was 300–600 mg/L, the total content of Pb and Zn in the roots of *S. argenteostriata* was 35,030.53 mg/kg on the 20th day, and the proportion of Pb and Zn was 92.49% and 7.51%, respectively. The results of this study can provide the basis for the application of *S. argenteostriata* in the remediation of the combined Pb–Zn contamination of soil. In the future, a long-term soil culture experiment should be conducted to further verify the toxicity of combined Pb–Zn stress on *S. argenteostriata* and the interaction between the elements.

## Figures and Tables

**Table 1:** Impacts of different treatment times and Zn treatments on the bioaccumulation factor in four organs. Z1, Z2, Z3, Z4 and Z5 indicate that the Zn concentrations are 75, 150, 300, 600 and 1000 mg/L, respectively.

Treatment Time	Zn Stress Treatment	Bioconcentration Factors			
		Root	Rhizome	Stem	Leaf
15d	Z1	85.2	34.9	19.4	5.6
	Z2	56.3	20.4	16.8	6.9
	Z3	35.0	10.8	8.4	4.7
	Z4	20.8	7.3	7.0	4.4
	Z5	11.2	4.7	4.1	4.1
45d	Z1	137.1	46.2	21.5	8.2
	Z2	70.2	16.2	17.6	9.9
	Z3	32.3	10.9	12.7	8.8
	Z4	29.8	9.4	7.1	6.7
	Z5	8.3	4.4	5.0	5.5

**Table 2:** Impacts of various Pb–Zn treatments on bioconcentration factors in four organs for 20 days. PZ0, PZ1, PZ2, PZ3 and PZ4 indicate that the Pb concentration is 300 mg/L and that the Zn concentrations are 0, 75, 150, 300 and 600 mg/L, respectively.

Organ Species	Pb–Zn Stress Treatment	Bioconcentration Factors		
		Pb	Zn	Total
Root	PZ0	18.0	-	-
	PZ1	38.7	34.8	37.9
	PZ2	77.5	27.4	60.8
	PZ3	80.3	25.3	52.8
	PZ4	108.0	4.4	38.9
Rhizome	PZ0	9.7	-	-
	PZ1	7.0	12.0	8.0
	PZ2	23.7	8.8	18.8
	PZ3	21.4	6.9	14.2
	PZ4	75.9	3.5	27.6
Stem	PZ0	3.5	-	-
	PZ1	3.4	6.7	4.1
	PZ2	7.2	7.2	7.2
	PZ3	9.2	6.8	8.0
	PZ4	33.1	4.9	14.3
Leaf	PZ0	1.9	-	-
	PZ1	2.2	2.6	2.3
	PZ2	8.0	2.3	6.1
	PZ3	9.4	2.8	6.1
	PZ4	8.0	4.1	5.4

Note: Because there was no Zn stress in the PZ0 treatment, the row was left blank.

## Data Availability

The authors declare that [the/all other] data supporting the findings of this study are available within the article.
